# Assessment of Sample Size Calculations Used in Aquaculture by Simulation Techniques

**DOI:** 10.3389/fvets.2020.00253

**Published:** 2020-05-19

**Authors:** Ignacio de Blas, Ana Muniesa, Adriana Vallejo, Imanol Ruiz-Zarzuela

**Affiliations:** ^1^Laboratory of Fish Diseases, Department of Animal Pathology, Instituto Agroalimentario de Aragón IA2, Universidad de Zaragoza, Zaragoza, Spain; ^2^Facultad de Medicina Veterinaria y Zootecnia, Universidad de Córdoba, Montería, Colombia

**Keywords:** sample size, random sampling, systematic sampling, cluster, infection detection, prevalence estimation

## Abstract

An adequate sampling methodology is the key to knowing the health status of aquatic populations. Usually, the aims of epidemiological surveys in aquaculture are to detect an infection and estimate the disease prevalence, and different formulas are used to calculate the sample size. The main objective of this study was to assess if the sample sizes calculated using classical epidemiological formulas are valid considering the sampling methodology, the population size, and the spatial distribution of diseased animals in the population (non-clustered or clustered). However, the use of sample sizes of 30, 60, and 150 fish is widely accepted in aquaculture, due to the requirements of the World Organization for Animal Health (OIE) for epidemiological surveillance. We have developed a specific software using ASP (*Active Server Pages*) language and MySQL database in order to generate aquatic populations from 100 to 10 000 brown trouts infected by *Aeromonas salmonicida* with different levels of prevalence: 2, 5, 10, and 50%. Then we implemented several Monte Carlo simulations to estimate empirically the sample sizes corresponding to the different scenarios. Furthermore, we compared these results with the values calculated by classical formulas. We determined that simple random sampling was more accurate in detecting an infection, because it is independent of the distribution of infected animals in the population. However, if diseased animals are non-clustered it is more efficient to use systematic methods, even in the case of small populations. Finally, the formula to calculate sample size to estimate disease prevalence is not valid when the expected prevalence is far from 50%, and it is necessary to increase the sample size to reach the desired precision.

## Introduction

Epidemiological surveillance in aquatic populations aims to assess the risk of the introduction and spreading of pathogens (1), however a balanced relationship cost-benefit is required.

One of the key elements of a surveillance program is the sampling method, and it should warrant the representativity of the results (2). The sample size varies considerably depending on the expected results, since the goals of surveillance are usually pathogen detection and prevalence estimation (3, 4).

The detection of a specific pathogen is the main objective of the surveillance programs for notifiable diseases (5), and in this case, the limiting factor is the collection of a sufficient number of samples (4). Generally, a non-probability sampling method is used, and the sample size is directly related to expected prevalence (design prevalence), so the higher the prevalence is, the more chance to find an infected animal, and the required sample size is lower (6).

The estimation of prevalence is also important in control and eradication programs to assess their effectivity based on the prevalence variation along time. In this case, it is necessary to use a probability sampling method to know the probability that a randomly selected animal of a population was infected in any specific moment of time (4).

In aquaculture, the use of sample sizes of 30, 60, and 150 fish is widely accepted, according to requirements of the OIE for epidemiological surveillance for detection of a disease with 2, 5, and 10% of minimum expected prevalence, respectively. The reason for these numbers is the wrong assumption that the population size is considered as “infinite” in the case of aquatic animals. However, in many surveys the most appropriate sample size should be calculated according to the population size and the objective of the study. In the case of populations of terrestrial animals, sample sizes are clearly related to population size and specific tables are usually provided to select the most adequate sample size for different scenarios and purposes (5).

Two basic elements should be considered: sampling method and sample size. On the one hand, the sampling method can be either non-probability or probability. Non-probability methods provide non-representative samples due to biases, and in some cases, their use could be interesting to increase the probability of finding animals with a specific feature (as in surveillance programs to detect pathogens). However, if the sample has to be representative of the population, the sampling method should be based on probability and individuals must be randomly collected using two basic methods: simple random sampling and systematic sampling. The former means any individual in the population has the same chance of being selected, and needs both a census with individual identification and a system to generate random numbers from this census. The latter is a more efficient method based on the collection of samples, taking into account the intervals when individuals in a population can be ordered (2–4).

In the case of fish populations, a non-probability method is used to detect a disease in case of mortalities and/or outbreaks, and fish with clinical signs are collected as samples to confirm the cause of the disease. This sample is not suitable to calculate the prevalence. So, the main challenge is to sample an asymptomatic population because a probability method must be used. As we commented previously, there are two main approaches: simple random and systematic. Simple random sampling is complex to use in fish populations because it requires the individual identification of each animal. Systematic sampling is possible during the transfer of animals (i.e., fish triage), as a certain number of fish can be collected, taking into account previously defined intervals. However, other strategies are possible in aquatic populations based on the selection of sampling points. In this case, the simple random sampling is based on random generation of coordinates in the pond, and the systematic sampling is based on the random selection of a point in the pond and the application of a grid to select consecutive sampling points.

On the other hand, the sample size depends on the objective of the study. For the detection of pathogens, the formula (1) calculates the sample size (*n*) that allows the detection of at least one infected animal from a population (with *N* animals) with an expected number of infected animals (*d*, that could be calculated as the product of minimum expected prevalence and population size) assuming a probability of 1-α (3).

(1) $$\begin{array}{r} n\;=\left(1-\alpha^{1/d}\right)\cdot (N-\frac{d-1}{2}). \end{array}$$

When we want to estimate the prevalence in an “infinite” population, the formula (2) is used to calculate the sample size (*n*) that depends on expected prevalence (*P*), accepted error (*E*), and confidence level (1- α, that determine the value of *Z* in a normal distribution) (3).

(2) $$\begin{array}{r} n={\left(Z_{\alpha /2}\frac{P(1-P)}{E}\right)}^{2}. \end{array}$$

However, the formula (2) does not consider population size and it is possible to obtain a sampling fraction (*n/N*), even >100%. In order to avoid a sampling with replacement, an adjusted sample size (*n*_*a*_) is calculated using the formula (3) when the sampling fraction is >10%, (2, 7). Usually, it is not necessary to adjust the sample size in a fish population because the population size is usually very large.

(3) $$\begin{array}{r} n_{a}=\frac{n}{1+\frac{n}{N}}=\frac{Nn}{N+n}=\frac{1}{\frac{1}{n}+\frac{1}{N}}. \end{array}$$

Taking into account the sampling methodology described above, which is commonly used in aquatic animal health, we propose an empirical verification of its validity using simulation methods.

## Materials and Methods

### Model Variables Description

Firstly, sampling will be carried out in a culture pond of brown trout (*Salmo trutta*) to detect infection and to calculate the prevalence of *Aeromonas salmonicida*, the etiological agent of the furunculosis. The prevalence of infection by *A. salmonicida* in asymptomatic trout populations is around 26% (8).

Population size will be from 100 to 10 000 fish, with intervals of 100 for a range between 100 and 1 000, 500 for a range between 1 000 and 5 000, and 1 000 for a range between 5 000 and 10 000. So, populations of 23 different sizes were generated and located in a pond of 400 ×400 cm. The size of the pond is used only for simulation purposes because the sample size is independent of population density. For the prevalence of infection, we generated these populations with four different prevalences (2, 5, 10, and 50%). Lower prevalences (2, 5, and 10%) were used to detect the pathogen and higher prevalences (5, 10, and 50%) to estimate the prevalence. The distributions of infected trout were random (non-clustered) and grouped in 1, 3, or 5 clusters of different sizes.

### Simulation of Populations

**Figure 1:**
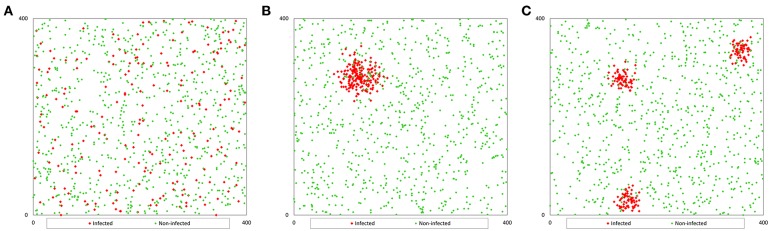
Example of the spatial distribution of a population of 1 000 fish with a prevalence of 20%: **(A)** non-clustered distribution; **(B)** clustered distribution (1 cluster); **(C)** clustered distribution (3 clusters).

To generate a population with a random distribution of infection (Pr), we randomly distributed each fish in the pond, generating two coordinates (*x, y*) by using a function that generates random numbers between 0 and 1, and multiplying the value by 400 to adjust to the pond size (Figure 1A). We consider a two-dimensional space instead of a three-dimensional space to locate (for further collection) the fish because the selection of sampling points is carried out based on the surface of the pond (independently of depth).

However, to generate the population with clustered infection (Pc), we considered that infected fish were grouped in 1, 3, or 5 clusters with the same number of infected fish in each cluster. We generated coordinates for the first infected fish for each cluster (*x*_0_*, y*_0_) and the coordinates of the rest of the infected fish in each cluster were generated assuming a random variable normally distributed in a radius of 400 divided by 20 times the square root of the number of clusters (*c*). This value was determined by trial and error method. The coordinates of the infected fish were calculated with the formula (4) that uses random numbers between 0 and 1 (RND) (9), and the coordinates of non-infected fish were calculated as population Pr (Figures 1B,C).

(4) $$\begin{array}{r} x\;=\;x_{0}+\frac{400}{20\cdot \sqrt{c}}\cdot \sqrt{-2\cdot log\left(RND\right)}\cdot cos\left(2\cdot \pi \cdot RND\right).\\ y\;=y_{0}+\frac{400}{20\cdot \sqrt{c}}\cdot \sqrt{-2\cdot log\left(RND\right)}\cdot \text{cos}\left(2\cdot \pi \cdot RND\right). \end{array}$$

### Simulation of Sampling Procedures

The next step was to define two sampling methods without replacement: simple random and repeated systematic sampling, using in both cases individual samples. The algorithm for simple random sampling was very simple because we assumed individual identification from 1 to population size and random numbers were generated in the interval [1, *N*] to select fish.

**Figure 2:**
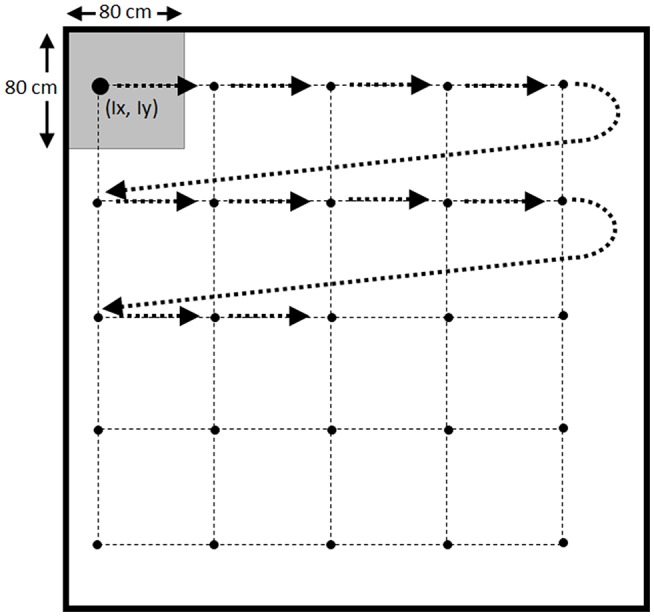
Scheme for the repeated systematic sampling using a grid of 5x5 points.

Repeated systematic sampling was based on the location of fish in the pond, combined with the use of a grid with 5 × 5 points separated by 80 cm. The upper-left corner of the grid was randomly located in a coordinate (*I*_*x*_*, I*_*y*_) inside a square of 80 × 80 cm located in the upper-left corner of the pond (Figure 2). Samples were collected in the order shown in Figure 2, catching the fish closer to each point of the grid according to Pythagoras theorem (10). The grid was relocated again until the target of sampling was reached (11).

Considering the number of options used for different variables of the model [the infection distributions (*n* = 4), the infection prevalences (*n* = 3), the population sizes (*n* = 23), and the sampling methods (*n* = 2)] a total of 552 different scenarios were simulated (Table 1).

**Table 1:** Description of factors simulated in the sampling model.

**Factor**	**Options**
Population size	100, 200, 300, 500, 500, 600, 700, 800, 900, 1 000, 1 500, 2 000, 2 500, 3 000, 3 500, 4 000, 4 500, 5 000, 6 000, 7 000, 8 000, 9 000, 10 000
Distribution of infection	Non-clustered, 1 cluster, 3 clusters, 5 clusters
Prevalence of infection	SRS (2%, 5%, 10%), RSS (5%, 10%, 50%)
Sampling method	Simple random (SRS), repeated systematic (RSS)

Simulations using Monte Carlo method can be used to obtain approximated numeric solutions to quantitative problems, with or without certainty (12). This method combines statistical concepts with the capacity of computers to generate pseudo-random numbers and to automatize calculations using algorithms (2, 4). It is especially useful to solve complex problems where an analytical approach is difficult or impossible to obtain.

In the detect infection scenario, the samples were collected until the first infected trout was selected, while in the estimate prevalence scenario, the samples were collected until two requirements were met: calculated error (*E*) was equal or lower than 0.05 [calculated as formula (5), derived from formulas (2) and (3)], and prevalence of infection of the population was into the interval [*P - E, P* + *E*] (where *P* is the calculated prevalence using the collected sample).

(5) $$\begin{array}{r} E\;=\;Z_{\frac{\alpha }{2}}\cdot \sqrt{\frac{P\cdot \left(1-P\right)}{\frac{n\cdot N}{n+N}}}. \end{array}$$

For each model, 10 different populations were simulated with 500 iterations. The sample size was the percentile 95, meaning, the minimum sample size that allows for obtaining of the sampling target in 95% of iterations. The average of these 10 results was used for further analysis and comparison with sample sizes calculated with formulas (1), (2), and (3).

### Software to Implement Models

Algorithms used to generate populations and to simulate samplings were implemented with ASP 3.0 language (programming language for websites based on Microsoft Visual Basic) using a web server based on Microsoft IIS (*Internet Information Services*). For data management, a database implemented with MySQL 4.5 was used. Database tables and source code are available to researchers upon request. Finally, calculations and plots were carried out using Microsoft Excel 2016.

## Results

### Sampling to Detect Infection

#### Simple Random Sampling

**Figure 3:**
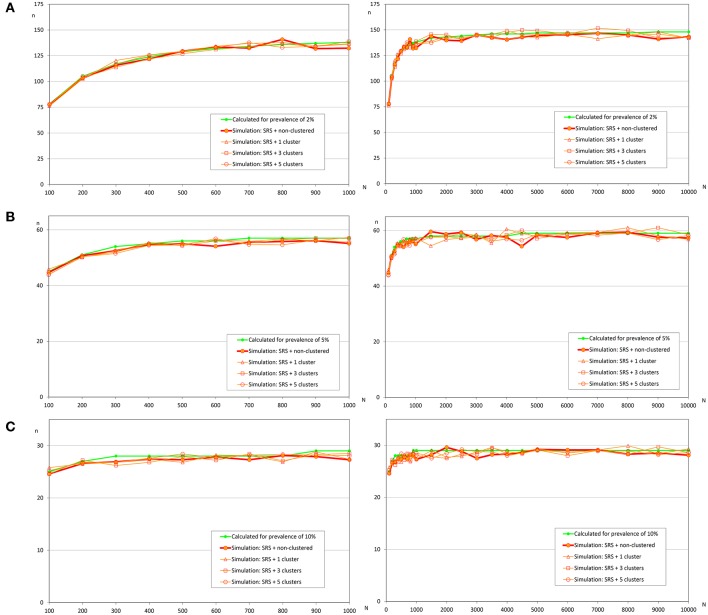
Sample sizes to detect an infection using simple random sampling. **(A)** Prevalence of infection = 2%; **(B)** Prevalence of infection = 5%; **(C)** Prevalence of infection = 10%.

Table 2 shows the relative differences between sample sizes calculated with the formula (1) and that were obtained by simulation. So a positive value indicates that sample size estimated by simulation is greater than the sample size calculated with the formula (it means than the assessed formula underestimates the required sample size); on the other hand, a negative value indicates that sample size estimated by simulation is lower than the sample size calculated with the formula (it means than the assessed formula overestimates the required sample size). Independently of the prevalence of infection, the spatial distribution of infected fish, and population size, the simple random sampling allows detection of at least one infected fish using the sample size calculated with formula (1). The samples sizes obtained by simulation were lower than 2% compared with them (Figure 3).

**Table 2:** Average relative deviation of sample size for infection detection obtained by simulation compared with sample size calculated with formula (1) using simple random sampling.

**Simple Random Sampling**		**Minimum expected prevalence**		
		**2%**	**5%**	**10%**
Distribution of infected fish	Non-clustered	−1.5%	−1.4%	−1.9%
	1 cluster	−1.0%	−0.8%	−1.2%
	3 clusters	−0.4%	−0.8%	−1.8%
	5 clusters	−0.6%	−1.6%	−1.2%

#### Repeated Systematic Sampling

When a repeated systematic sampling was carried out to detect an infection in a population, we observed that the sample size calculated in a population with a non-clustered distribution of infected fish was slightly lower than that obtained by simulation. However, the repeated systematic sampling was not efficient when infected fish were clustered, and the needed sample size was increased with the prevalence of infection and the number of clusters (Table 3).

**Table 3:** Average relative deviation of sample size for infection detection obtained by simulation compared with sample size calculated with formula (1) using repeated systematic sampling.

**Repeated Systematic Sampling**		**Minimum expected prevalence**		
		**2%**	**5%**	**10%**
Distribution of infected fish	Non–clustered	−3.9%	−4.4%	−8.0%
	1 cluster	40.4%	77.4%	143.6%
	3 clusters	39.2%	91.0%	160.1%
	5 clusters	37.1%	66.3%	155.2%

**Figure 4:**
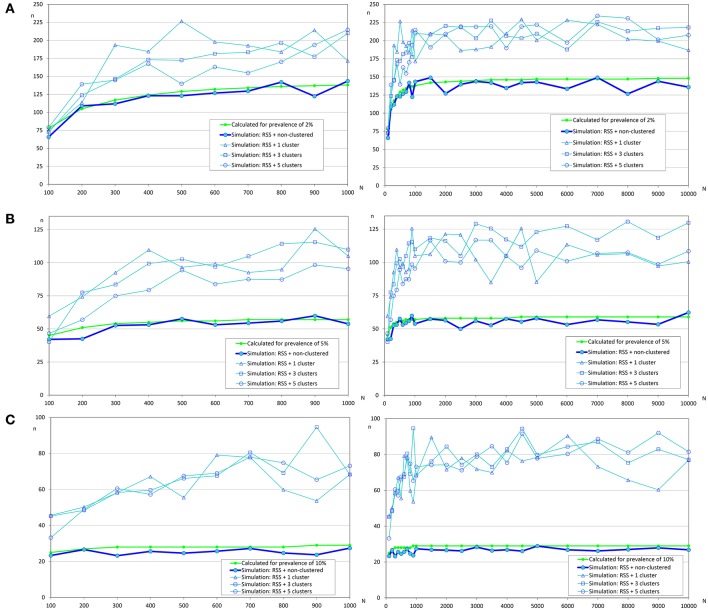
Sample sizes to detect an infection using repeated systematic sampling. **(A)** Prevalence of infection = 2%; **(B)** Prevalence of infection = 5%; **(C)** Prevalence of infection = 10%.

In Figure 4, it can be observed that sample sizes were increased when repeated systematic sampling was carried out, and this increment was directly related to the prevalence of infection. It was interesting that in small populations the population size was also directly correlated with simulated sample size, but in populations >1 000 fish the sample sizes tend to be constant [similar to the asymptotic behavior of formula (1)].

Finally, the effect of the number of clusters on the sample size was not clear and apparently the increment of sample size was lower when there were more clusters. Further studies will be needed to evaluate the influence of this variable on the sample size.

### Sampling to Estimate the Prevalence

#### Simple Random Sampling

**Figure 5:**
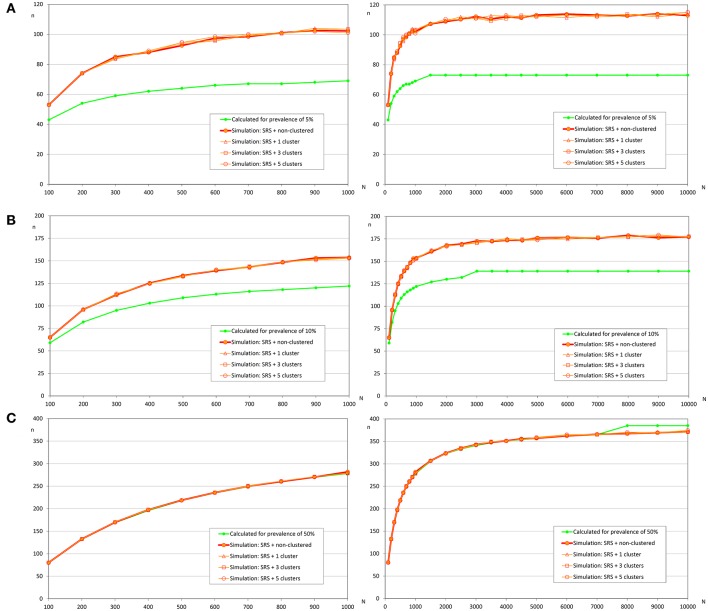
Sample sizes to estimate a prevalence using simple random sampling. **(A)** Prevalence of infection = 5%, and accepted error = 5%; **(B)** Prevalence of infection = 10%, and accepted error = 5%; **(C)** Prevalence of infection = 50%, and accepted error = 5%.

Similar to the scenario to detect an infection, we did not find relevant differences according to the distribution of the infected fish (Table 4 and Figure 5). However, the expected prevalence had a great influence in the sample size calculated by simulation, and when prevalence was close to 50% there were no differences between the calculated and the simulated sample sizes. But when the expected prevalence was far from 50%, the sample sizes calculated by simulation were greater.

**Table 4:** Average relative deviation of sample size for prevalence estimation obtained by simulation compared with sample size calculated with formulas (3) and (4) using simple random sampling (assuming accepted error of 5%).

**Simple Random Sampling**		**Expected prevalence**		
		**5%**	**10%**	**50%**
Distribution of infected fish	Non–clustered	48.9%	24.4%	−0.2%
	1 cluster	49.0%	24.3%	−0.3%
	3 clusters	49.0%	24.3%	−0.2%
	5 clusters	49.1%	24.2%	−0.2%

#### Repeated Systematic Sampling

Firstly, we must indicate that it was necessary to modify the conditions of simulations to obtain the result of this section for the scenarios with prevalences of 10 and 50%. Modification was also necessary with clustered infection due to the server being unable to complete the foreseen iterations, the modified conditions were the population size (only 100, 250, 500, 1 000, 2 500, and 5 000 fish), and the reduction to four simulated populations by iteration.

**Figure 6:**
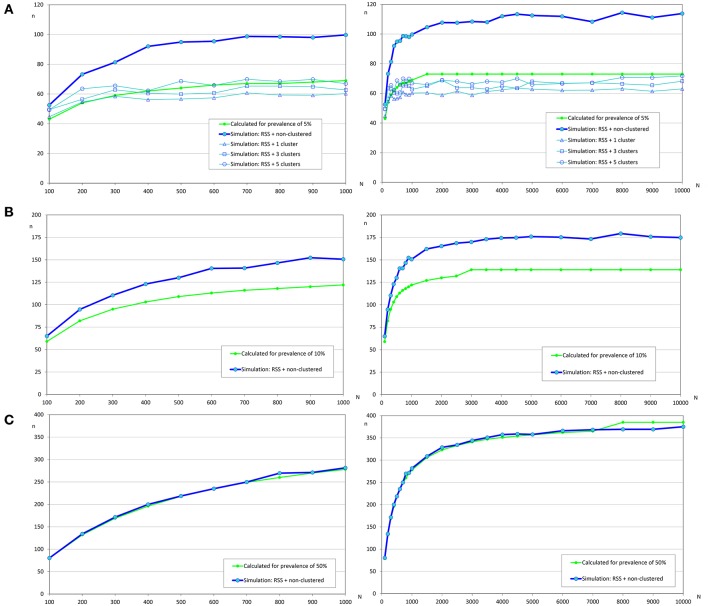
Sample sizes to estimate a prevalence using repeated systematic sampling. **(A)** Prevalence of infection = 5%, and accepted error = 5%; **(B)** Prevalence of infection = 10%, and accepted error = 5%; **(C)** Prevalence of infection = 50%, and accepted error = 5%.

The variation of sample sizes for repeated systematic sampling was similar to the results corresponding to the simple random sampling when infection was randomly distributed (Table 5 and Figure 6). However, the sample size calculated by simulation was lower than the theoretical value calculated with formulas (3) and (4), but only when prevalence was low (5%). The reduction of sample sizes was greater when the number of clusters was increased.

**Table 5:** Average relative deviation of sample size for prevalence estimation obtained by simulation compared with sample size calculated with formulas (3) and (4) using repeated systematic sampling (assuming accepted error of 5%).

**Repeated Systematic Sampling**		**Expected prevalence**		
		**5%**	**10%**	**50%**
Distribution of infected fish	Non–clustered	47.1%	23.5%	0.5%
	1 cluster	−12.1%	573.5%	337.5%
	3 clusters	−6.1%	484.7%	340.5%
	5 clusters	−1.2%	313.2%	340.6%

**Figure 7:**
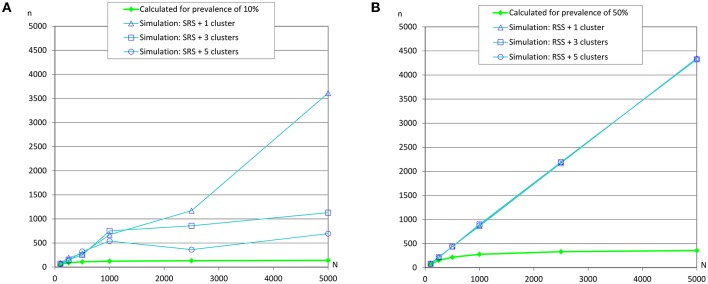
Sample sizes to estimate a prevalence using repeated systematic sampling. **(A)** Prevalence of infection = 10%, and accepted error = 5%; **(B)** Prevalence of infection = 50%, and accepted error = 5%.

However, simulations collapsed the server when the prevalences were 10 and 50% (due to overflow of the database because the simulation did not meet the required conditions to finish), and as we previously commented, simpler simulations were carried out (Table 5 and Figure 7) and the required sample sizes by simulation were much greater than the calculated values. It could be due to the dimensions of the grid and the number of clusters.

## Discussion

The OIE recommends some generic sample sizes to detect infected fish in a population (5, 13). These values are very frequent in epidemiological texts (150 individuals to detect infection over 2%, 60 for 5%, and 30 for 10%) and are derived directly from the formula (1).

These sample sizes are suitable for detecting infection when a simple random sampling is used, independently of the prevalence and distribution of infected fish, according to results obtained by simulation, so simple random sampling provides us a representative sample. However, the repeated systematic sampling worked better, but only when the infected fish were randomly distributed, and it did not work when infected fish were clustered. The relative increment of simulated sample size was greater with medium prevalence (10%), but it was because the calculated sample size is small (n≈30). In any case, the simulated sample sizes were greater for lower prevalences, and apparently, the number of clusters did not affect to sample size. Some authors have described that a systematic sampling was more inefficient than a simple random sampling (7, 14); however, it is widely used in epidemiological surveys due to its operational simplicity (15).

Based on our results, we cannot recommend a repeated systematic sampling approach to detect an infection when we suspect the presence of clusters of infected animals, as a simple random sampling would be more efficient. Other similar works have been carried out using iterative procedures to calculate sample sizes in complex epidemiological scenarios (16, 17).

Related to the estimation of the prevalence, the main factor that affects the sample size was the expected prevalence. When the expected prevalence was 50%, the calculated sample sizes were almost identical to those obtained by simulation with a simple random sampling (independently of infection distribution) and with a repeated systematic sampling (only with random infection) (Figures 5, 6). But, when the prevalence of infection decreased, the simulated sample sizes increased.

These results agree with similar works. Williams et al. (18) estimated the sample size by simulation with Monte Carlo method to compare two proportions and it was necessary to use a sample size 38.5% greater than calculated with a traditional formula, especially in situations of low prevalence. In the same way, Newcombe and Soto (19) indicated that confidence intervals for a proportion with the formula (5) were not valid for proportions far from 50% and they suggested the use of Wilson's score method (formula 6) (20).

(6) $$\begin{array}{r} \frac{2nP+Z_{\alpha /2}^{2}\pm Z_{\alpha /2}\sqrt{4nP(1-\text{P})+Z_{\alpha /2}^{2}}}{2(n+Z_{\alpha /2}^{2})} \end{array}$$

Based on these considerations, Vallejo et al. (21) proposed a new formula to calculate the sample size to estimate a proportion with an algorithm checked by simulation, which corrected the deviations observed in this work.

Additionally, as in the case of infection detection, the repeated systematic sampling to estimate a prevalence was very inefficient compared with simple random sampling when there were clusters of infected animals.

Finally, a limitation of this study is that simulated populations are distributed uniformly in the pond, and further simulation should be carry out assuming irregular distributions, in separated groups (i.e., schools of fish) or with different densities of fish along the pond (i.e., concentration of animals in the center of the pond or in the area of outlet water).

## Conclusions

Simple random sampling is more efficient than repeated systematic sampling as it avoids bias due to a selection scheme based on a grid when populations have clusters of infection. The classical formula used to calculate the sample size to detect a pathogen in a fish population is valid in all simulated scenarios implemented, but the formula used to estimate the prevalence only works when expected prevalences are closer to 50%.

## Data Availability Statement

Raw data are not available due to the huge amount of information generated in each simulation. However, the code used to implement the model and create the database will be made available with undue reservation to any qualified researcher.

## Author Contributions

IB and AM: design of the article and development of simulations. IB and AV: implementation of graphs and figures. IR-Z: review of the writing of the article.

## Conflict of Interest

The authors declare that the research was conducted in the absence of any commercial or financial relationships that could be construed as a potential conflict of interest.
